# Associations between individual antipsychotics and the risk of arrests and convictions of violent and other crime: a nationwide within-individual study of 74 925 persons

**DOI:** 10.1017/S0033291721000556

**Published:** 2022-12

**Authors:** Amir Sariaslan, Stefan Leucht, Johan Zetterqvist, Paul Lichtenstein, Seena Fazel

**Affiliations:** 1 Department of Psychiatry, University of Oxford, Warneford Hospital, Oxford, UK; 2 Social and Public Policy Unit, Faculty of Social Sciences, University of Helsinki, Helsinki, Finland; 3 Department of Psychiatry and Psychotherapy, Technische Universität München, München, Germany; 4 Department of Psychosis Studies, Institute of Psychiatry, National Institute for Health Research, Mental Health Biomedical Research Centre, King's College London, London, UK; 5 Institute for Environmental Medicine, Karolinska Institutet, Stockholm, Sweden; 6 Department of Medical Epidemiology and Biostatistics, Karolinska Institutet, Stockholm, Sweden

**Keywords:** Antipsychotics, effectiveness, violence, crime, pharmacoepidemiology, clozapine

## Abstract

**Background:**

Individuals diagnosed with psychiatric disorders who are prescribed antipsychotics have lower rates of violence and crime but the differential effects of specific antipsychotics are not known. We investigated associations between 10 specific antipsychotic medications and subsequent risks for a range of criminal outcomes.

**Methods:**

We identified 74 925 individuals who were ever prescribed antipsychotics between 2006 and 2013 using nationwide Swedish registries. We tested for five specific first-generation antipsychotics (levomepromazine, perphenazine, haloperidol, flupentixol, and zuclopenthixol) and five second-generation antipsychotics (clozapine, olanzapine, quetiapine, risperidone, and aripiprazole). The outcomes included violent, drug-related, and any criminal arrests and convictions. We conducted within-individual analyses using fixed-effects Poisson regression models that compared rates of outcomes between periods when each individual was either on or off medication to account for time-stable unmeasured confounders. All models were adjusted for age and concurrent mood stabilizer medications.

**Results:**

The relative risks of all crime outcomes were substantially reduced [range of adjusted rate ratios (aRRs): 0.50–0.67] during periods when the patients were prescribed antipsychotics *v.* periods when they were not. We found that clozapine (aRRs: 0.28–0.44), olanzapine (aRRs: 0.46–0.72), and risperidone (aRRs: 0.53–0.64) were associated with lower arrest and conviction risks than other antipsychotics, including quetiapine (aRRs: 0.68–0.84) and haloperidol (aRRs: 0.67–0.77). Long-acting injectables as a combined medication class were associated with lower risks of the outcomes but only risperidone was associated with lower risks of all six outcomes (aRRs: 0.33–0.69).

**Conclusions:**

There is heterogeneity in the associations between specific antipsychotics and subsequent arrests and convictions for any drug-related and violent crimes.

Antipsychotics are widely used medications that reduce overall psychotic symptoms, hospitalization rates, and relapse prevention (Huhn et al., 2019), but there is weaker evidence for their effects on social outcomes (Leucht et al., 2012). One significant set of social outcomes are violence, antisocial behaviours, and crime, which are perpetrated by a small minority of persons with psychiatric disorders, but increased in certain high-risk populations such as first-episode psychosis (Tiihonen et al., 2019), individuals with schizophrenia-spectrum disorders with background criminal histories (Buchanan, Sint, Swanson, & Rosenheck, 2019; Sariaslan, Arseneault, Larsson, Lichtenstein, & Fazel, 2020; Stevens, Laursen, Mortensen, Agerbo, & Dean, 2015) and comorbid substance use disorders (Fazel, Långström, Hjern, Grann, & Lichtenstein, 2009; Kwan et al., 2018; Lamsma, Cahn, Fazel, & GROUP and NEDEN investigators, 2019; Sariaslan, Larsson, & Fazel, 2016), and people with trauma histories (Fitton, Yu, & Fazel, 2020; MacManus et al., 2012). These are important outcomes to prevent as their risk factors are partly modifiable and can interfere with mental health care if treatment is interrupted. Antipsychotics as an overall class of medications appear to reduce violent arrest and conviction risks, supported by trial data based on a small number of randomized controlled trials (Leucht et al., 2012), and a population within-individual population study of violent and any crime outcomes (Fazel, Zetterqvist, Larsson, Långström, & Lichtenstein, 2014). However, importantly, it is not known as to which specific antipsychotics are most effective at reducing violence and criminal risk, apart from clozapine that is associated with clear reductions in criminal conviction risk (Bhavsar et al., 2019) but whose use is highly restricted.

Clarifying the gradient of effectiveness for antipsychotics is important to inform clinical decision-making in view of many different antipsychotics in oral and intramuscular forms (Kahn et al., 2018), and to understand underlying mechanisms of the links between disorders and outcomes. Clinical trials are not feasible for crime outcomes as follow-up periods are not sufficiently long, and trials not adequately powered (Zhu et al., 2017). Furthermore, many high-risk patients are excluded from entering and hence trials may not provide generalizability. Thus, observational studies provide an alternative approach to examine antipsychotic effectiveness in real-world settings, particularly if confounding by indication can be accounted for (Chang et al., 2019).

In this investigation of nearly 75 000 people who were prescribed antipsychotics, we have used high-quality Swedish national registers to examine associations of a wide range of antipsychotics with crime outcomes, including violent arrest, and used a within-individual design so that outcomes are measured in the same person when they are prescribed a particular medication compared to when they are not. This approach allowed us to indirectly account for all unmeasured time-constant factors within each individual (e.g. genetic and childhood environmental factors) (Frisell, Lichtenstein, & Långström, 2011; Kowalec et al., 2019; Lichtenstein et al., 2006) and to examine how diagnostic categories (e.g. patients with and without psychotic disorders) moderated the associations.

## Methods

### Study design and patients

The Swedish government provides all residents a personal identification number, which is used to accurately link many routinely collected registers (Ludvigsson et al., 2016). Following an approval from the regional research ethics committee of Karolinska Institutet (2013/5:8), we were given access to de-identified data from Statistics Sweden, which we used to study all individuals born in Sweden between 1961 and 1990 (2 240 557 men and 2 128 205 women). This means all of the participants had reached the age of legal responsibility in Sweden (15 years) at the start of the follow-up in July 2005. We then identified a subset of individuals from this population sample who have ever been prescribed antipsychotics or mood stabilizers according to the Swedish Prescribed Drug Register. This register includes information about all prescribed and dispensed medication since July 2005 (*<*0.3% of entries had missing patient identity data) (Wettermark et al., 2007). The data on arrests and convictions were identified from the National Crime Register, which includes all arrests and convictions in Sweden since 1973 (Sariaslan et al., 2016). We additionally identified dates of emigrations and deaths via the Migration and Causes of Death registers, to account for actual time at risk for crime outcomes. Periods in prison and psychiatric hospitals were estimated using the Prison Register and the National Patient Register, the latter of which including for all psychiatric hospital admissions since 1973 (and for outpatient care since 2001) (Ludvigsson et al., 2011).

### Definitions and measures

We extracted data about treatment with antipsychotics and mood stabilizers, identified in the Swedish Prescribed Drug Register according to the anatomical therapeutic chemical (ATC) classification system. Antipsychotics were defined as drugs with ATC codes N05A, excluding lithium (N05AN01). We included the following specific first-generation antipsychotics: levomepromazine (N05AA02), perphenazine (N05AB03), haloperidol (N05AD01), flupentixol (N05AF01), zuclopenthixol (N05AF05) and second-generation antipsychotics: clozapine (N05AH02), olanzapine (N05AH03), quetiapine (N05AH04), risperidone (N05AX08), and aripiprazole (N05AX12). These specific drugs were selected as they were sufficiently prevalent in our sample (*>*2%). We also accounted for less prevalent antipsychotics by creating two combined classes of ‘other first-generation antipsychotics’ (5.6%) and ‘other second-generation antipsychotics’ (6.6%). Mood stabilizers were defined as valproic acid or sodium valproate (N03AG01), lamotrigine (N03AX09), carbamazepine (N03AF01), oxcarbazepine (N03AF02), or lithium. Long-term-acting injectables (LAIs) were identified as injections of any antipsychotic medication which are typically administered in intervals of between 2 and 4 weeks (Correll et al., 2016). In separate analyses, we also examined the following specific LAIs: perphenazine, haloperidol, flupentixol, zuclopenthixol and risperidone. For descriptive purposes, we also examined prescription rates of antidepressants (N06A), hypnotics/anxiolytics (N05B, N05C), stimulants (N06BA), and medications used to treat substance use disorders (N07B).

The start of treatment was defined as the date of the first prescription, and end of treatment as the date of the final prescription during. These dates are the days on which the prescriptions were collected. A patient was defined to be receiving treatment during the time interval between two dispensed prescriptions of medication, unless prescriptions were issued more than 122 days (4 months) apart. We based this decision because oral medications are unlikely to be dispensed for more than 90 days at a time in Swedish routine psychiatric practice (Sveriges Riksdag [Swedish Parliament], 2002). We therefore defined a treatment period as a sequence of at least two prescriptions, with a maximum of 4 months between any two consecutive prescriptions. If such periods exceeded 4 months, we categorized the individual to be off treatment. Since the Prescribed Drug Register covered the period 1 July 2005–31 December 2013, the start of follow-up was set as 1 January 2006 to establish whether the participants were receiving treatment at the start of the follow-up period (i.e. at least 4 months after the register started). The end of the follow-up was set to 31 December 2013, because we only had register data until this point. Consistent with previous work, we excluded time periods when the individuals were in prison or psychiatric hospitals from the follow-up as the data on prescriptions is likely to be unreliable and there would be less opportunity for criminal behaviour, which would also be underreported, during these specific periods.

### Diagnostic categories

From the National Patient Register, we identified individuals diagnosed with any psychotic disorders, which included schizophrenia-spectrum disorders, bipolar disorders, or other psychotic disorders (including depressive psychosis, and drug-induced psychoses; ICD-codes are presented in Online Supplementary Table S1). Diagnostic validity is typically good to high for psychiatric disorders in the National Patient Register: schizophrenia (concordance rates of 86% in comparisons with file reviews by psychiatrists) (Ekholm et al., 2005), bipolar disorder (concordance of 92%) (Sellgren, Landén, Lichtenstein, Hultman, & Långström, 2011), depression (concordance of 88%) (Fazel et al., 2015), anxiety disorders (positive predictive values: 86%–97%) (Rück et al., 2015), personality disorders (concordance of 93%) (Kouppis & Ekselius, 2020), and substance use disorders (concordance of 68%) (Fazel et al., 2009).

### Outcomes

The primary outcomes were criminal arrests for violent crime (e.g. homicide, assault, robbery, arson, any sexual offence, illegal threats, or intimidation) (Sariaslan et al., 2016), drug-related crime and any crime. We also investigated convictions for crime. In separate analyses, to test the extent to which drug-related and other crime outcomes were associated with substance misuse, we examined acute substance intoxication, resulting in either hospitalization or death, as a secondary outcome.

### Statistical analyses

We initially fitted a series of standard Poisson regression models to the full sample of individuals who had been prescribed with antipsychotic for any treatment period at least once throughout the 8 years of follow-up (*n* = 74 925). This model captured between-individual associations: comparisons of the crime rates between individuals who were on antipsychotic medication *v.* those who were off medication. Medication status was included in the models as a time-varying covariate. The between-individual analyses were adjusted for sex, age (continuous, in years), and concurrent mood stabilizer medications. The latter two covariates were allowed to vary across time. To account for unmeasured time-constant individual-level confounders, we subsequently fitted Poisson regression models with a fixed-effects estimator, where we entered each individual as a unique stratum. This allows for comparison of crime rates within each individual (e.g. during periods on and off medications in the same person) rather than between individuals. Given these comparisons, the model requires that each individual has at least one period of being unexposed to antipsychotics during the follow-up period. Approximately 7% (*n* = 5582) of the sample were excluded from these analyses as they were continuously prescribed antipsychotics throughout the follow-up period. The within-individual models were adjusted for age and concurrent mood stabilizer medications as time-varying covariates. As the fixed-effects estimator only focuses on within-individual variation, by its design, it indirectly accounts for an aggregate of all factors that do not change throughout the follow-up period, whether these are measured or not (Allison, 2009; Kaufman, 2008). Consequently, we did not adjust for any time-invariant confounders, whether they were measured (e.g. sex and criminal background at the baseline) or not (e.g. genetic background, childhood environmental influences and pre-baseline antipsychotic prescriptions) as they were accounted for by the statistical model. Therefore, we did not directly measure and adjust for familial factors. We used cluster-robust standard errors in all of the models to account for correlations between periods within the same individuals. It should be noted that our approach contrasts with the single-case treatment design framework, which primarily rely on visual methods (Lane & Gast, 2014) and statistical models that do not account for unmeasured confounding (DeHart & Kaplan, 2019; Manolov & Onghena, 2018) to assess treatment effects.

To test for the relative contributions of each specific oral antipsychotic medication on the risk for violent crime arrests, we fitted a within-individual model where we included each of the 10 antipsychotic medications as well as the two pooled measures of other first- and second-generation antipsychotics as time-varying covariates. The individuals who were previously excluded from the within-individual analyses for being continuously prescribed to antipsychotics throughout the follow-up (*n* = 5582) contributed instead to these analyses as they had been prescribed at least two different types of antipsychotics. We adjusted the model for other concurrent LAIs and mood stabilizer prescriptions. We assessed the effects of the medications across all crime outcomes by calculating the sum of their rank for each outcome. We conducted similar analyses for specific LAIs where we adjusted for concurrent mood stabilizer prescriptions. In the latter analyses, we included all LAIs that had a prevalence rate of over one percent in the sample.

### Sensitivity analyses

To establish whether the reported associations could be explained by selection effects and test the robustness of our findings, we initially stratified the main analyses across sub-samples of men and women, and also by 10-year birth cohorts (e.g. those born between 1961–1970, 1971–1980, and 1981–1990). We further tested whether our findings were moderated by diagnostic category (psychotic disorders *v.* not), pre-baseline history of criminal offending, and being prescribed antipsychotics at the baseline of the study by re-running the main within-individual analyses in these specific subsets of the sample. We additionally tested for moderation by psychotic disorders for the individual antipsychotics but restricted the outcomes to any and violent crime arrests to maximize the statistical power of these sensitivity analyses. As the effects of LAIs can last up to six months after they are administered, we re-ran the analyses for the LAIs by testing for a more conservative definition of non-medicated periods of two consecutive prescriptions being issued for more than 1 year apart. Finally, we explored to what extent reverse causation bias may have contributed by excluding crime events that had occurred between 7 and 90 days prior to the start of antipsychotic prescription.

## Results

**Fig. 1.**
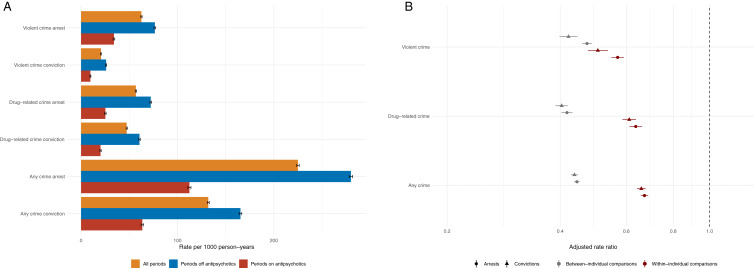
Absolute (*A*) and relative (*B*) risks of violent crime, drug-related crime and any crime convictions and arrests across periods on and off prescriptions to antipsychotics among 74 925 individuals who were ever prescribed antipsychotics between 2006 and 2013 in Sweden. *Notes*: The between-individual comparisons are based on Poisson regression models that were adjusted for sex and age. The within-individual comparisons are based on fixed-effects Poisson regression models that were adjusted for age and concurrent mood stabilizer medications.

In Swedes who were born between 1961 and 1990 (*n*_men_ = 2 240 557; *n*_women_ = 2 128 205), we identified 37 565 (1.7%) men and 37 360 (1.8%) women who were prescribed with any antipsychotic between 1 January 2006 and 31 December 2013. The baseline characteristics of these patients are presented in Table 1 and the distribution of prescriptions of individual antipsychotics during the follow-up across patients with and without psychotic disorders are presented in Table 2. During the study period, 8815 men (23.5%) were arrested for 25 559 violent crimes, and 3271 women (8.8%) were arrested for 6719 violent crimes, in this cohort. The unadjusted rates for violent crime arrests and convictions were considerably lower during periods when the individuals were prescribed antipsychotics (9.7–34.1 events per 1000 person-years; Fig. 1*A*; Online Supplementary Table S2) as compared to periods when they were not (26.0–76.6 events per 1000 person-years; Fig. 1*A*; Online Supplementary Table S2). We found similar results for any and drug-related arrests, and when using convictions instead of arrests for all outcomes (Fig. 1*A*; Online Supplementary Table S2).

**Table 1.** Background characteristics of individuals prescribed with antipsychotics in Sweden, 2006–2013

	Men	Women
*Total*		
Individuals	37 565	37 360
Person-years at risk (mean)	253 346 (6.7)	262 679 (7.0)
*Sociodemographic factors measured in 2006*		
Age group (years)		
15–24	9575 (25.5%)	9601 (25.7%)
24–39	18 315 (48.8%)	18 160 (48.6%)
≥40	9675 (25.8%)	9599 (25.7%)
Civil status		
Unknown	1151 (3.1%)	1023 (2.7%)
Unmarried	29 356 (78.1%)	24 362 (65.2%)
Married	4504 (12.0%)	7400 (19.8%)
Divorced	2519 (6.7%)	4469 (12.0%)
Widowed	35 (0.1%)	106 (0.3%)
Living in metropolitan area	5731 (15.3%)	5541 (14.8%)
Employed	11 927 (31.8%)	12 960 (34.7%)
Studying	5623 (15.0%)	7681 (20.6%)
Median family-adjusted income, US$ (inter-quartile range)	15 102 (11 333-18 884)	14 004 (10 384-17 203)
*Lifetime psychiatric disorders*		
Any psychotic disorder	21 785 (58.0%)	20 399 (54.6%)
Schizophrenia	7263 (19.3%)	3713 (9.9%)
Bipolar disorder	5313 (14.1%)	9217 (24.7%)
Other psychotic disorder	9209 (24.5%)	7469 (20.0%)
Depression	7714 (20.5%)	10 240 (27.4%)
Antisocial personality disorder	899 (2.4%)	229 (0.6%)
Other personality disorders	6748 (18.0%)	10 599 (28.4%)
Alcohol use disorder	8791 (23.4%)	6319 (16.9%)
Drug use disorder	10 805 (28.8%)	7980 (21.4%)

*Note*: The table includes individuals in the cohort who had more than one disorder. The main classes of psychiatric disorders were defined hierarchically across schizophrenia, bipolar disorder, other psychotic disorder and depression. The personality disorders (e.g. antisocial and other personality disorders) and substance use disorders (alcohol and drug use disorders) were allowed to be comorbid with the main classes of psychiatric disorders.

**Table 2.** Antipsychotics and other medications prescribed during the follow-up period (2006–2013)

	No psychotic disorders	Any psychotic disorder
*Any antipsychotic medication*	32 741 (100%)	42 184 (100%)
*First-generation antipsychotics*		
Levomepromazine	5027 (15.4%)	5674 (13.5%)
Perphenazine	562 (1.7%)	2035 (4.8%)
Haloperidol	1088 (3.3%)	3057 (7.2%)
Flupentixol	2356 (7.2%)	1768 (4.2%)
Zuclopenthixol	613 (1.9%)	2623 (6.2%)
Other first-generation antipsychotics	2831 (8.6%)	1393 (3.3%)
*Second-generation antipsychotics*		
Clozapine	208 (0.6%)	3520 (8.3%)
Olanzapine	8804 (26.9%)	19 786 (46.9%)
Quetiapine	10 266 (31.4%)	14 411 (34.2%)
Risperidone	5963 (18.2%)	9574 (22.7%)
Aripiprazole	3234 (9.9%)	10 453 (24.8%)
Other second-generation antipsychotics	1070 (3.3%)	3880 (9.2%)
*Other medications*		
Mood stabilizers	7000 (21.4%)	17 425 (41.3%)
Antidepressants	29 153 (89.0%)	31 695 (75.1%)
Hypnotics/anxiolytics	29 476 (90.0%)	37 331 (88.5%)
Stimulants	5843 (17.8%)	5414 (12.8%)
Drugs used in addictive disorders	6159 (18.8%)	7492 (17.8%)

To examine the relative risks, we initially performed between-individual analyses where we compared rates of violent arrests during medication periods with non-medication periods in a cohort of 74 925 patients who had at least one period on medication during follow-up (Fig. 1*B*; Online Supplementary Table S3). We found that periods of antipsychotic prescriptions were associated with a 53% rate of reduction in violent arrests [adjusted rate ratio (aRR): 0.47, 95% confidence interval (CI): 0.46–0.49]. The magnitude of the associations for the other crime outcomes were similar, ranging from 56% reduction for any crime arrests (aRR: 0.44, 95% CI: 0.44–0.45) to 60% reduction for drug-related convictions (aRR: 0.40, 95% CI: 0.39–0.42).

**Fig. 2.**
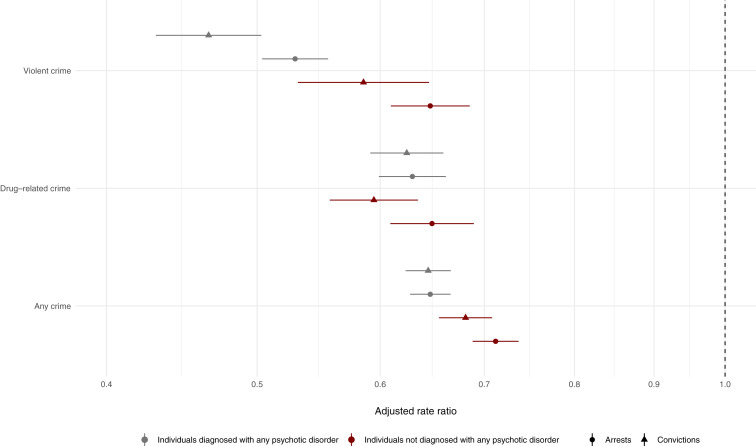
Within-individual associations between antipsychotic prescriptions and criminal outcomes (violent crime, drug-related crime and any crime arrests and convictions) stratified across people with and without psychotic disorders. *Notes:* The within-individual comparisons are based on fixed-effects Poisson regression models that were adjusted for age and concurrent mood stabilizer medications.

By comparing violent arrest risks within each individual across time, during periods when they were on and off antipsychotics, we were able to account for all time-constant unmeasured factors (e.g. genetics and shared childhood environments). We found that periods of antipsychotic prescriptions were associated with a 43% rate reduction (aRR: 0.57, 95% CI: 0.55–0.59; Fig. 1*B*, Online Supplementary Table S3). Similar associations were observed for other outcomes, ranging from a 33% rate reduction for any crime arrests (aRR: 0.67, 95% CI: 0.66–0.69) to a 50% rate reduction for violent crime convictions (aRR: 0.50, 95% CI: 0.47–0.54). When we stratified these within-individual associations across psychotic disorders, we found that the magnitude of the associations with violent crime arrests was stronger in persons diagnosed with psychotic disorders (aRR: 0.53; 95% CI: 0.50–0.56; Fig. 2, Online Supplementary Table S4) than those without psychotic disorders (aRR: 0.65, 95% CI: 0.61–0.69; Fig. 2, Online Supplementary Table S4). We found similar results for violent crime convictions and any crime arrests as outcome (Fig. 2; Online Supplementary Table S4).

**Fig. 3.**
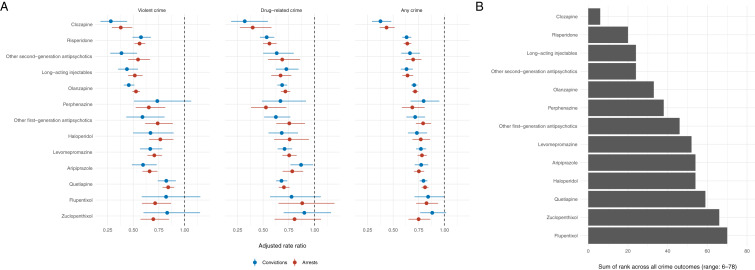
Within-individual associations between prescriptions of specific antipsychotic medications and criminal outcomes (violent, drug-related and any crime arrest) (*A*) and their summative rank across all six criminal outcomes (*B*). *Notes*: The within-individual comparisons are based on fixed-effects Poisson regression models that were adjusted for age and concurrent mood stabilizer medications.

We subsequently examined associations between specific oral antipsychotics and the six crime outcomes using within-individual comparisons (Fig. 3*A*; Online Supplementary Table S5). All medications were associated with lower rates of violent crime arrests but there was substantial heterogeneity in the estimates, which ranged from clozapine (aRR: 0.38, 95% CI%: 0.29–0.49) to quetiapine (aRR: 0.84, 95% CI: 0.79–0.90). The estimates further varied across the three different types of crime (e.g. violent, drug-related, and any) but typically to a lesser extent between arrests and convictions within each crime type (Fig. 3*A*; Online Supplementary Table S4). We found that the within-individual associations between the specific oral antipsychotics on violent and any crime arrests tended to be stronger among patients diagnosed with psychotic disorders relative to patients without such conditions (Online Supplementary Figure S1). The findings for clozapine in non-psychotic patient groups were less clear, partly because only 0.6% (*n* = 208; Table 2) of these patients had been prescribed clozapine during the follow-up period. When we ranked the medications across all six crime outcomes and summed these to create a summary ranking, clozapine, risperidone, olanzapine, and LAIs were associated with lower rates of criminality than other antipsychotic medications (Fig. 3*B*). We additionally examined equivalent estimates of specific LAIs (Online Supplementary Figure S2). Despite limited statistical power, we found that risperidone was associated with clearly lower rates of violent crime arrests than haloperidol, zuclopenthixol, perphenazine, or flupentixol.

In complementary sensitivity analyses, we found no material differences to the presented findings when we stratified the associations across men and women (Online Supplementary Figure S3), 10-year birth cohorts (Online Supplementary Figure S4), pre-baseline history of criminal offending (Online Supplementary Figure S5), and being prescribed antipsychotics at the baseline of the study (Online Supplementary Figure S6). We further found commensurate results by examining acute substance intoxication as the outcome (aRR_between-individual_: 0.86, 95% CI: 0.80–0.92; aRR_within-individual_: 0.91, 95% CI: 0.83–0.99). To account for the potential bias induced by reverse causation (e.g. a violent arrest leading to a new antipsychotic prescription), we excluded violent crime arrests that had occurred within 7 up to 90 days before every medication period. The differences between the estimates were negligible (Online Supplementary Figure S7). For the specific LAIs, we did not find any material differences when we extended the definition of non-medicated periods from 4 months to 1 year between two consecutive prescriptions (Online Supplementary Figure S8).

## Discussion

In this population-based study of 74 925 individuals prescribed with antipsychotics and who were followed for up to 8 years, we examined the associations of individual antipsychotics for risk of arrest for violent, drug-related and other crimes. We used a within-individual design so that the same person was examined for arrest risk when they were dispensed with antipsychotics compared to when they were not, which accounted for time-invariant factors, such as genetic and early environmental background factors. We report three principal findings.

First, we found clear differences in the associations of individual antipsychotics and violent crime risks. Clozapine, olanzapine, risperidone, and long-acting injectable antipsychotics were associated with larger reductions in violent arrest rates than some other commonly prescribed oral antipsychotics such as quetiapine and haloperidol. This heterogeneity was also found within long-acting injectables, with risperidone being associated with stronger reductions than other common long-acting injectables. This is a potentially important finding as the real-world effectiveness of long-acting antipsychotics has been an area of uncertainty (Correll, Rubio, & Kane, 2018; Kishimoto et al., 2018).

Second, we estimated that periods of antipsychotic prescriptions were associated with a 43% lower risk of being for violent offences when the comparisons were made within individuals. Although a replication of a previous population-based study (Fazel et al., 2014), this investigation has a considerably larger sample, a more sensitive outcome of arrest, and stratification by sex.

We initially found large sex differences in the absolute risks of the crime outcomes, which is a widely replicated finding in the criminological and psychological literature (Choy, Raine, Venables, & Farrington, 2017). However, we did not find that sex differences moderated the relative risks of the outcomes when we stratified the fully adjusted within-individual models by sex. In addition, we examined individual antipsychotics for the first time. Furthermore, we investigated two other crime outcomes – drug-related and any crime – and found that antipsychotics were also associated with lower risks, but less strongly than for violent criminality. These findings underscore the real-world effectiveness of antipsychotics for a broad range of outcomes.

Third, in relation to drug-related arrest and arrest for any crime, which are more common than violent arrest and can also disrupt psychiatric care, we found that a similar pattern of heterogeneity of the effects of individual antipsychotics, with clozapine, risperidone, and long-acting antipsychotics having stronger associations than other antipsychotics. This information is novel to our knowledge. One possible reason why long-acting antipsychotics were associated with reduced risks of crime outcomes is that they are dispensed by health care staff, and typically for those with more severe symptoms, which means that assessments of mental state can be made during administration of medication, and additional treatment instituted.

The current findings of individual antipsychotics should be seen in the context of the findings of clinical trials (Huhn et al., 2019). A recent meta-analysis found that clozapine was associated with the greatest reductions in symptom scores, and also that olanzapine and risperidone were among the more effective medications for symptomatic relief (Huhn et al., 2019). For social functioning, the network meta-analysis was unable to investigate clozapine and was not sufficiently powered to determine differences. In contrast, our investigation was able to study a range of crime outcomes, which contributes to disruption in health and social care, stigma, costs and morbidity to patients, their carers, and potential victims. In addition, a recent large observational study in Sweden on people with schizophrenia, using within-individual models, with hospitalization as the main outcome also reported that contrasting associations with individual antipsychotics, with clozapine, long-acting injectables, and olanzapine being more effective than quetiapine (Tiihonen et al., 2019). In contrast, the current study was not limited to schizophrenia, and we included individuals without psychotic disorders who represented over 40% of the cohort. This reflects the real-world practice of the wider use of antipsychotics for symptomatic relief in other mental disorders (such as personality disorders) and in people without formal diagnoses, and also more specific indications in other severe mental illnesses (such as bipolar disorder and moderate/severe depression). The current findings therefore provide real-world evidence to assist clinicians to decide which antipsychotics to use.

The overlap of the associations of some individual antipsychotics between trials (where the outcomes are symptom scores and hospitalization) and real-world outcomes (such as registered crime in the current report) suggests that psychotic symptoms may potentially be one mechanism for crime outcomes. This was further supported by our findings demonstrating that these associations were stronger in patients with psychotic disorders as compared to patients with non-psychotic disorders. Risk factor studies support overall symptoms scores being a predictor of violence (Witt, van Dorn, & Fazel, 2013). In addition, it has also been proposed that clozapine has a specific anti-aggressive effect (Meltzer et al., 2003), supported in subsequent trials (Frogley, Taylor, Dickens, & Picchioni, 2012), which is postulated to be a consequence of its stronger binding to a subtype of serotonin receptors (Meltzer, 1994). This particular receptor-binding effect is shared with olanzapine, albeit to a lesser degree. Another possible mechanism is that the antipsychotics reduce substance misuse comorbidity, and we found support for this in a sensitivity analysis that hospital contacts for intoxication were reduced in people dispensed antipsychotics. Substance intoxication has previously been found to act as a trigger for violent episodes in people diagnosed with psychotic disorders (and in the general population) (Sariaslan, Lichtenstein, Larsson, & Fazel, 2016). Finally, antipsychotic use may be a marker of more support by health care professionals on a range of interpersonal and social issues, which will be complicated by the direction of these effects. The explanation for the current findings might be a combination of these, although more psychosocial support may be particularly strong in clozapine due to regular monitoring that accompanies its prescription. This is unlikely to the sole explanation as regular monitoring is a feature of long-acting injectables, which we found had less strong crime-reducing effects.

The strengths of our study include the use of the Swedish nationwide registry data, which enabled us to study 74 925 individuals who were prescribed antipsychotics between 2006 and 2013 with minimal selection bias, given the universal health care system of Sweden. In contrast to previous studies that have generally relied on conviction data, we additionally examined arrests. This approach is more comprehensive because it complements the limitations of both measures (e.g. not all people who commit crimes are convicted and not all arrested people have committed a crime). In relation to generalizability, Sweden has similar rates of mental illness (Wittchen et al., 2011), rates of antipsychotic prescription (Fazel et al., 2014), and rates of violent assault (Heiskanen, 2010) than other European countries and North America. Although some particular crime rates, such as homicide, are different to those in the US, these remain rare from a population crime perspective, and their contribution to overall morbidity is considerably less than any crime and all violent crimes.

The within-individual design offered a powerful approach to account for unmeasured confounding. The differences observed across the different specific medications within each crime type in addition to the relatively stable pattern of associations between the crime types demonstrated the value of this methodological approach.

However, some limitations are noted. First, while the within-individual design accounts for all time-stable individual-level unmeasured confounders, the estimates may potentially be biased by time-varying confounders, such as changes in socioeconomic status, social networks, and substance use comorbidity (Sariaslan, Larsson, Lichtenstein, & Fazel, 2017). However, it remains unlikely that such factors would explain the differences observed between different specific antipsychotics. In addition, an earlier Swedish study found that a comprehensive set of socioeconomic status indicators did not confound the within-individual association between clozapine prescriptions and violent crime convictions (Bhavsar et al., 2019). Second, the prescription drug register data only covers data on prescriptions that are dispensed and collected, and we do not know whether individuals actually took their medication. However, this possible misclassification will have biased our estimates downward as medication non-adherence is a strong risk factor for violence (Rezansoff, Moniruzzaman, Fazel, McCandless, & Somers, 2017; Witt et al., 2013). This is further supported by trial data (Leucht et al., 2012) and the fact that we obtained similar results for the long-acting injectable antipsychotics. Finally, our findings might be partially explained by reverse causation, where violent and other criminal actions increase the likelihood of the patients being prescribed antipsychotic medications. However, we did not find evidence of such biases when we conducted a series of sensitivity tests by excluding crime events having occurred up to three months prior to each antipsychotic dispensation. Although unlikely, it remains a possibility that less severe acts of aggression and antisocial behaviors that do not result in an arrest could potentially bias the reported associations. Large-scale clinical studies are therefore warranted to investigate this possibility.

In conclusion, we found clear heterogeneity in the effects of specific antipsychotics for these real-world and clinically important crime outcomes. These findings may assist in developing more precise and personalized treatments for individuals prescribed antipsychotics.

## Supplementary material

For supplementary material accompanying this paper visit https://doi.org/10.1017/S0033291721000556. click here to view supplementary material
