# Supplementary material for: Associations between individual antipsychotics and the risk of arrests and convictions of violent and other crime: a nationwide within-individual study of 74 925 persons
Source: Psychol Med. 2021 Mar 11;52(16):3792–800. doi: 10.1017/S0033291721000556 (PMC9811342; doi:10.1017/S0033291721000556)
Supplement: Supplementary file 1 [file S0033291721000556sup001.docx]

**eTable 1. ICD codes**

|  | **ICD-8**  **(1973-1986)** | **ICD-9**  **(1987-1996)** | **ICD-10**  **(1997-)** | **Minimum number of episodes** |
| --- | --- | --- | --- | --- |
| Schizophrenia | 295.0–6, 295.8, 295.9 | 295A–G, 295W, 295X | F20 | 2 |
| Bipolar disorder | 296.1, 296.3, 296.8, 296.9 | 296A, 296C-296E, 296W, 296X | F30-F31 | 2 |
| Other psychotic disorder | 291, 297, 298, 294.3 or 296.2 | 291, 292, 297-299, 295H or 296B | F10.5-F19.5, F21-F25, F28-F29, F32.3 or F33.3 | 1 |
| Depression | 300.4 | 300E, 311 | F32-F39 | 1 |
| Antisocial personality disorder | 301.7 | 301H | F60.2 | 1 |
| Other personality disorders | 301 (excl. 301.7) | 301 (excl. 301H) | F60-F62, F69 (excl. F60.2) | 1 |
| Alcohol use disorder | 303 | 303, 305A | F10 (excl. F10.5) | 1 |
| Drug use disorder | 304 | 304, 305X | F11-F19 (excl. F1*.5) | 1 |
| Acute substance intoxication | - | - | F10.0-F12.0, F14.0-F16.0, F19.0 | 1 |

**eTable 2. Absolute risks expressed as rates per 1000 person-years of violent crime, drug-related crime and any crime convictions and arrests across periods on and off prescriptions to antipsychotics among 75,204 individuals who were ever prescribed antipsychotics between 2006-2013 in Sweden**

|  | **Violent crime** | **Drug-related crime** | **Any crime** |
| --- | --- | --- | --- |
|  | **Rate per 1000 person-years**  **[95% CI]** | **Rate per 1000 person-years**  **[95% CI]** | **Rate per 1000 person-years**  **[95% CI]** |
| ***Arrests*** |  |  |  |
| All periods | 62.6 [61.9-63.3] | 56.8 [56.2-57.5] | 225.0 [223.7-226.3] |
| Periods off antipsychotics | 76.6 [75.6-77.5] | 72.3 [71.4-73.2] | 279.9 [278.2-281.7] |
| Periods on antipsychotics | 34.1 [33.2-35.0] | 25.2 [24.5-26.0] | 112.4 [110.8-114.0] |
|  |  |  |  |
| ***Convictions*** |  |  |  |
| All periods | 20.7 [20.3-21.1] | 47.4 [46.8-47.9] | 132.0 [131.0-133.0] |
| Periods off antipsychotics | 26.0 [25.5-26.6] | 60.6 [59.8-61.5] | 165.4 [164.0-166.7] |
| Periods on antipsychotics | 9.7 [9.2-10.2] | 20.2 [19.5-20.8] | 63.5 [62.3-64.7] |

**eTable 3. Relative risks expressed as adjusted rate ratios (aRRs) of violent crime, drug-related crime and any crime convictions and arrests across periods on and off prescriptions to antipsychotics among 75,204 individuals who were ever prescribed antipsychotics between 2006-2013 in Sweden**

|  | **Violent crime** | **Drug-related crime** | **Any crime** |
| --- | --- | --- | --- |
|  | **aRR [95% CI]** | **aRR [95% CI]** | **aRR [95% CI]** |
| ***Arrests*** |  |  |  |
| Between-individual comparisons | 0.47 [0.46-0.49] | 0.41 [0.40-0.43] | 0.44 [0.44-0.45] |
| Within-individual comparisons | 0.57 [0.55-0.59] | 0.64 [0.61-0.66] | 0.67 [0.66-0.69] |
|  |  |  |  |
| ***Convictions*** |  |  |  |
| Between-individual comparisons | 0.42 [0.40-0.44] | 0.40 [0.39-0.42] | 0.43 [0.43-0.45] |
| Within-individual comparisons | 0.50 [0.47-0.54] | 0.61 [0.59-0.64] | 0.66 [0.64-0.67] |

*Notes: The between-individual comparisons are based on Poisson regression models that are adjusted for sex and age. The within-individual comparisons are based on fixed-effects Poisson regression models that are adjusted for age. Concurrent mood stabiliser medications were adjusted for across both models. The nested data structure (e.g., periods nested within individuals) is accounted for through cluster-robust standard errors.*

**eTable 4. Within-individual associations between prescriptions of antipsychotic medications and criminal outcomes (violent crime, drug-related crime and any crime arrests and convictions) stratified across patients with and without psychotic disorders**

|  | **Violent crime** | **Drug-related crime** | **Any crime** |
| --- | --- | --- | --- |
|  | **aRR [95% CI]** |  | **aRR [95% CI]** |
| ***Arrest*** |  |  |  |
| No psychotic disorder | 0.65 [0.61-0.69] | 0.65 [0.61-0.69] | 0.71 [0.69-0.74] |
| Psychotic disorder | 0.53 [0.50-0.56] | 0.63 [0.60-0.66] | 0.65 [0.63-0.67] |
| ***Conviction*** |  |  |  |
| No psychotic disorder | 0.59 [0.53-0.65] | 0.59 [0.56-0.63] | 0.68 [0.65-0.71] |
| Psychotic disorder | 0.47 [0.43-0.50] | 0.62 [0.59-0.66] | 0.64 [0.62-0.67] |

*Notes: The within-individual comparisons are based on fixed-effects Poisson regression models that are adjusted for age. The nested data structure (e.g., periods nested within individuals) is accounted for through cluster-robust standard errors. Concurrent mood stabilizer medications were adjusted for.*

**eTable 5. Within-individual associations between prescriptions of specific antipsychotic medications and criminal outcomes (violent, drug-related and any crime arrest)**

|  | **Violent crime** | **Drug-related crime** | **Any crime** |
| --- | --- | --- | --- |
|  | **aRR [95% CI]** | **aRR [95% CI]** | **aRR [95% CI]** |
| ***Clozapine*** |  |  |  |
| Arrest | 0.38 [0.29-0.49] | 0.40 [0.27-0.58] | 0.44 [0.37-0.52] |
| Conviction | 0.28 [0.18-0.44] | 0.32 [0.19-0.55] | 0.38 [0.29-0.48] |
| ***Risperidone*** |  |  |  |
| Arrest | 0.56 [0.51-0.62] | 0.56 [0.50-0.63] | 0.64 [0.60-0.68] |
| Conviction | 0.58 [0.49-0.67] | 0.53 [0.47-0.61] | 0.63 [0.59-0.68] |
| ***Other second-generation antipsychotics*** |  |  |  |
| Arrest | 0.55 [0.45-0.66] | 0.69 [0.55-0.86] | 0.70 [0.62-0.78] |
| Conviction | 0.38 [0.28-0.54] | 0.63 [0.50-0.80] | 0.66 [0.58-0.76] |
| ***Long-acting injectables*** |  |  |  |
| Arrest | 0.52 [0.45-0.59] | 0.67 [0.58-0.77] | 0.64 [0.59-0.70] |
| Conviction | 0.44 [0.35-0.55] | 0.73 [0.62-0.85] | 0.63 [0.58-0.69] |
| ***Olanzapine*** |  |  |  |
| Arrest | 0.53 [0.49-0.56] | 0.72 [0.67-0.76] | 0.72 [0.69-0.75] |
| Conviction | 0.46 [0.41-0.51] | 0.69 [0.64-0.73] | 0.71 [0.68-0.74] |
| ***Perphenazine*** |  |  |  |
| Arrest | 0.65 [0.52-0.81] | 0.53 [0.38-0.73] | 0.69 [0.59-0.81] |
| Conviction | 0.74 [0.51-1.07] | 0.67 [0.49-0.92] | 0.80 [0.67-0.95] |
| ***Other first-generation antipsychotics*** |  |  |  |
| Arrest | 0.74 [0.62-0.89] | 0.76 [0.63-0.91] | 0.79 [0.72-0.87] |
| Conviction | 0.59 [0.43-0.81] | 0.62 [0.51-0.76] | 0.72 [0.63-0.81] |
| ***Haloperidol*** |  |  |  |
| Arrest | 0.76 [0.66-0.89] | 0.76 [0.61-0.95] | 0.77 [0.69-0.86] |
| Conviction | 0.67 [0.50-0.90] | 0.68 [0.55-0.84] | 0.73 [0.64-0.83] |
| ***Levomepromazine*** |  |  |  |
| Arrest | 0.71 [0.64-0.78] | 0.75 [0.69-0.83] | 0.78 [0.74-0.83] |
| Conviction | 0.67 [0.57-0.78] | 0.71 [0.64-0.78] | 0.77 [0.72-0.82] |
| ***Aripiprazole*** |  |  |  |
| Arrest | 0.66 [0.59-0.74] | 0.78 [0.69-0.89] | 0.75 [0.70-0.80] |
| Conviction | 0.60 [0.49-0.73] | 0.87 [0.77-0.99] | 0.77 [0.71-0.84] |
| ***Quetiapine*** |  |  |  |
| Arrest | 0.84 [0.79-0.90] | 0.70 [0.65-0.76] | 0.81 [0.78-0.85] |
| Conviction | 0.82 [0.74-0.92] | 0.68 [0.63-0.74] | 0.80 [0.76-0.84] |
| ***Flupentixol*** |  |  |  |
| Arrest | 0.71 [0.59-0.87] | 0.88 [0.65-1.20] | 0.83 [0.73-0.94] |
| Conviction | 0.82 [0.58-1.16] | 0.78 [0.57-1.06] | 0.84 [0.71-1.00] |
| ***Zuclopenthixol*** |  |  |  |
| Arrest | 0.70 [0.57-0.85] | 0.81 [0.61-1.06] | 0.75 [0.65-0.86] |
| Conviction | 0.83 [0.60-1.15] | 0.90 [0.70-1.16] | 0.88 [0.76-1.02] |

*Notes: The within-individual comparisons are based on fixed-effects Poisson regression models that are adjusted for age. The nested data structure (e.g., periods nested within individuals) is accounted for through cluster-robust standard errors. Concurrent mood stabilizer medications were adjusted for.*

**eFigure 1. Sensitivity test: Within-individual associations between individual antipsychotic prescriptions and criminal outcomes (violent crime and any crime arrests) stratified across people with and without psychotic disorders**

*Notes: The within-individual comparisons are based on fixed-effects Poisson regression models that are adjusted for age. The nested data structure (e.g., periods nested within individuals) is accounted for through cluster-robust standard errors. Concurrent mood stabiliser medications were adjusted for.*

**eFigure 2. Within-individual associations between prescriptions of specific long-term acting injectable antipsychotic medications and criminal outcomes (violent crime, drug-related crime and any crime arrests and convictions; A) as well as their rank across all six of the outcomes (B)**

*Notes: The within-individual comparisons are based on fixed-effects Poisson regression models that are adjusted for age. The nested data structure (e.g., periods nested within individuals) is accounted for through cluster-robust standard errors. Concurrent mood stabiliser medications were adjusted for.*

**eFigure 3. Sensitivity test: Sex-specific within-individual associations between prescriptions of antipsychotic medications and criminal outcomes (violent crime, drug-related crime and any crime arrests and convictions)**

*Notes: The within-individual comparisons are based on fixed-effects Poisson regression models that are adjusted for age. The nested data structure (e.g., periods nested within individuals) is accounted for through cluster-robust standard errors. Concurrent mood stabiliser medications were adjusted for.*

**eFigure 4. Sensitivity test: Within-individual associations between prescriptions of antipsychotic medications and criminal outcomes (violent crime, drug-related crime and any crime arrests and convictions) stratified across cohorts born in 1961-1970, 1971-1980 and 1981-1990**

*Notes: The within-individual comparisons are based on fixed-effects Poisson regression models that are adjusted for age. The nested data structure (e.g., periods nested within individuals) is accounted for through cluster-robust standard errors. Concurrent mood stabiliser medications were adjusted for.*

**eFigure 5. Sensitivity test: Within-individual associations between prescriptions of antipsychotic medications and criminal outcomes (violent crime, drug-related crime and any crime arrests and convictions) stratified across individuals who had a history of the specific crime outcomes prior to the baseline of the study**

*Notes: The within-individual comparisons are based on fixed-effects Poisson regression models that are adjusted for age. The nested data structure (e.g., periods nested within individuals) is accounted for through cluster-robust standard errors. Concurrent mood stabiliser medications were adjusted for.*

**eFigure 6. Sensitivity test: Within-individual associations between prescriptions of antipsychotic medications and criminal outcomes (violent crime, drug-related crime and any crime arrests and convictions) stratified across individuals with and without an antipsychotic medication prescription at the baseline**

*Notes: The within-individual comparisons are based on fixed-effects Poisson regression models that are adjusted for age. The nested data structure (e.g., periods nested within individuals) is accounted for through cluster-robust standard errors. Concurrent mood stabiliser medications were adjusted for.*

**eFigure 7. Sensitivity test: Within-individual associations between prescriptions of antipsychotic medications and criminal outcomes (violent crime, drug-related crime and any crime arrests and convictions) where the crime outcomes occurring between 7 to 90 days before the antipsychotic exposure were excluded**

*Notes: The within-individual comparisons are based on fixed-effects Poisson regression models that are adjusted for age. The nested data structure (e.g., periods nested within individuals) is accounted for through cluster-robust standard errors. Concurrent mood stabiliser medications were adjusted for.*

**eFigure 8. Sensitivity test: Within-individual associations between prescriptions of specific long-term acting injectable antipsychotic medications and criminal outcomes (violent crime, drug-related crime and any crime arrests and convictions using two different definitions of non-medicated periods (4 months [reference] vs. 1 year)**

*Notes: The within-individual comparisons are based on fixed-effects Poisson regression models that are adjusted for age. The nested data structure (e.g., periods nested within individuals) is accounted for through cluster-robust standard errors. Concurrent mood stabiliser medications were adjusted for.*
